# Decorating unoxidized-carbon nanotubes with homogeneous Ni-Co spinel nanocrystals show superior performance for oxygen evolution/reduction reactions

**DOI:** 10.1038/srep45384

**Published:** 2017-03-30

**Authors:** Jun Yang, Tsuyohiko Fujigaya, Naotoshi Nakashima

**Affiliations:** 1 International Institute for Carbon-Neutral Energy Research (I2CNER), Kyushu University, 744 Motooka, Nishi-ku, Fukuoka 819-0395, Japan; 2 Department of Applied Chemistry, Graduate School of Engineering, Kyushu University, 744 Motooka, Nishi-ku, Fukuoka 819-0395, Japan; 3 PRESTO, JST, 4-1-8 Honcho, Kawaguchi, Saitama, 332-0012, Japan

## Abstract

We present a new concept for homogeneous spinel nanocrystal-coating on high crystalline pristine-carbon nanotubes (CNTs) for efficient and durable oxygen evolution reaction (OER) and oxygen reduction reaction (ORR). Oxidized CNTs have widely been used to functionalize with metal or metal oxides since the defect sites act as anchoring for metal oxide binding. However, such defects on the tubes cause the decrease in electrical conductivity and stability, leading to lower catalyst performance. In the present study, at first, pristine multi-walled carbon nanotubes (MWNTs) were wrapped by pyridine-based polybenzimidazole (PyPBI) to which uniform Ni_*x*_Co_3−*x*_O_4_ nanocrystals were homogeneously deposited by the solvothermal method without damaging the MWNTs, in which PyPBI acted as efficient anchoring sites for the deposition of spinel oxide nanocrystals with ~5 nm size. The obtained catalyst (MWNT-PyPBI-Ni_*x*_Co_3−*x*_O_4_) outperformed most state-of-the-art non-precious metal-based bifunctional catalysts; namely, for OER, the potential at 10 mA cm^−2^ and Tafel slope in 1 M KOH solution were 1.54 V vs. RHE and 42 mV dec^−1^, respectively. For ORR, the onset and half-wave potentials are 0.918 V and 0.811 V vs. RHE, respectively. Moreover, the MWNT-PyPBI-Ni_*x*_Co_3−*x*_O_4_ demonstrates an excellent durability for both ORR and OER.

The explosively increasing demands for portable electronic devices, electric vehicles, and efficient utilization of excess electricity and renewable energies have significantly stimulated the development of electrochemical energy storage systems, such as fuel cells, redox fuels, supercapacitors, rechargeable batteries, etc ref. 1. One of the key challenges in many energy conversion systems is to develop an efficient air electrode, in which the oxygen reduction reaction (ORR) or oxygen evolution reaction (OER) occur with very sluggish kinetics^2^,^3^. In particular, efficient and durable bifunctional electrocatalysts for both the ORR and OER are of significant importance to lower the overpotentials and improve the energy conversion efficiency of rechargeable metal-air batteries and reversible fuel cells^4^. Precious metal-based catalysts, such as nanoparticles of Pt and IrO_2_ have been well known as excellent electrocatalysts for the ORR and OER, respectively^5^,^6^. Considering the cost and source scarcity, however, non-precious metal catalysts from earth-abundant elements are highly desired from industry. In view of this, extensive efforts have been made to develop efficient ORR/OER bifunctional catalysts by using transition metal oxides and sulfides, doped nanocarbons, etc refs 4,7,8, while the performance of previously-reported catalysts are not very efficient.

Cobalt-based spinel oxides have been intensively studied as efficient bifunctional catalysts in alkaline and neutral media^9^,^10^,^11^. By incorporating Ni into A-site, NiCo_2_O_4_ possesses a higher electrical conductivity and more electrochemically active sites than pure Co_3_O_4_, leading to an enhanced activity^12^. However, the performance of NiCo_2_O_4_ is limited due to its insufficient electrical conductivity^13^,^14^. Loading these transition metal oxides on support materials with high electrical conductivity and large surface area is an effective way to overcome this drawback. Conventional carbon materials, such as carbon black, are conductive and low cost. Unfortunately, they are in a practical sense not suitable support materials for OER, which occurs at potentials higher than 1.23 V vs. RHE, because the electro-oxidation of carbon shown in eq1 has a theoretical onset potential as low as 0.207 V vs. RHE^15^.


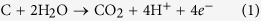


Highly crystallized graphitic surfaces of multi-walled carbon nanotubes (MWNTs) provide a superior electrochemical stability compared to other conductive carbons^16^,^17^,^18^ because the oxidation potential of the sp^2^ carbon is much higher than those of the sp^3^ carbons^15^. Moreover, MWNTs possess very high electrical conductivity and are easy to form freestanding electrode film. However, pristine MWNTs are in lack of binding sites on their surfaces. Therefore, it is necessary to create defects on the surfaces of MWNTs by oxidizing treatment prior to hybridizing with metal oxides^12^. Such a pretreatment turns a large proportion of sp^2^ carbon of the MWNTs to sp^3^ carbon and consequently decreases their electrical conductivity and stability of the catalyst^19^. Thus far, no report has been published describing homogeneous deposition of a metal oxide on the pristine CNTs.

Here we present a new concept for homogeneous spinel nanocrystal-coating on pristine-MWNTs. Our method is to use pyridine-based polybenzimidazole (PyPBI)-wrapped MWNTs to which uniform Ni_*x*_Co_3−*x*_O_4_ nanoparticles were successfully deposited by the solvothermal method without damaging the MWNTs (Fig. 1). In the catalyst, a thin layer of PyPBI on the MWNTs acts as an efficient anchoring layer for many different kinds of cations including Pt, Au, and Pd on pristine MWNTs^20^,^21^,^22^,^23^,^24^,^25^,^26^,^27^. We report the first successful application of this strategy to metal oxides and found that uniform Ni_*x*_Co_3−*x*_O_4_ nanoparticles were homogeneously deposited on the PyPBI-wrapped MWNTs without causing any damage to the MWNTs. The obtained catalyst, which is hereafter denoted as MWNT-PyPBI-Ni_*x*_Co_3−*x*_O_4_, was found to exhibit an excellent electrocatalytic activity and stability for both oxygen reduction reaction (ORR) and oxygen evolution reaction (OER). Experimental procedures of the synthesis of MWNT-PyPBI-Ni_*x*_Co_3−*x*_O_4_ is described in the Supplementary Information. For comparison, nickel hydroxide or cobalt oxide was also deposited on the MWNT-PyPBI separately by a similar method to MWNT-PyPBI-Ni_*x*_Co_3−*x*_O_4_. The resultant Ni- and Co-containing products are denoted as MWNT-PyPBI-Ni(OH)_2_ and MWNT-PyPBI-Co_3_O_4_, respectively. According to the thermogravimetric analysis (TGA) results, the weight ratios of metal oxides in MWNT-PyPBI-Ni_*x*_Co_3−*x*_O_4_, MWNT-PyPBI-Ni(OH)_2_, and MWNT-PyPBI-Co_3_O_4_ were estimated to be 37 wt%, 40 wt%, 39 wt%, respectively. MWNTs were wrapped by PyPBI by a facile method as explained elsewhere, and the weight ratio of PyPBI was determined to be *ca*. 6 wt% by thermogravimetric analysis^26^.

As shown in Fig. 2a, the MWNTs were coated with an extremely thin layer of PyPBI. The content of N on the surface of MWNT-PyPBI was estimated to be ~2.2%. Since the thickness of the PyPBI layer is less than 1 nm, we consider that the PyPBI would not interrupt the electron transfer between catalyst and the MWNT support^20^,^21^,^22^,^23^,^24^,^25^,^26^,^27^. Representative morphologies of the prepared catalysts are shown in Fig. 2b–h (scanning transmission electron microscopy (STEM) images at lower magnification are shown in Supplementary Information, Figure S1). As can be seen in Fig. 2b, uniform Ni_*x*_Co_3−*x*_O_4_ nanoparticles were homogeneously deposited on the MWNT-PyPBI after the solvothermal synthesis. The energy dispersed spectroscopy analysis (EDS) results have revealed that these nanoparticles are composed of Co and Ni, and the atomic ratio of Ni to Co was estimated to be 0.43 (see Figure S1 and Table S1). The lattice space values of the nanoparticles were assigned to the planes of the spinel oxide as shown in Fig. 2c.

These results indicated that the Ni_*x*_Co_3−*x*_O_*y*_ spinel oxide nanoparticles were *in-situ* formed on the MWNT-PyPBI. The PyPBI layer played a key role for the formation of such a fine homogeneous Ni_*x*_Co_3−*x*_O_4_ nanostructure on the MWNT-PyPBI because in the absence of PyPBI, only large aggregates (~150 nm) were formed on the pristine MWNTs after the same synthesis procedure (see Supplementary Information, Figure S3). This is because the bare surfaces of pristine MWNTs lack binding sites of metal oxides. In sharp contrast to MWNT-PyPBI-Ni_*x*_Co_3−*x*_O_4_, we see large aggregates (~150 nm) for the prepared MWNT-PyPBI-Co_3_O_4_ (Fig. 2d,e), which were composed of smaller nanoparticles with the (311) faces of the Co_3_O_4_ spinel oxide. A similar agglomeration of Co_3_O_4_ nanoparticles on un-oxidized MWNTs was also reported by Liu *et al*. and explained by the easy formation and agglomeration of the cobalt spinel oxide nuclei during the hydrothermal treatment^28^. For MWNT-PyPBI-Ni(OH)_2_, a large amount of nanosized β-Ni(OH)_2_ particles with the indexed (011) faces (Fig. 2f) were recognized on the MWNT-PyPBI. Figure 2g shows that the particle size of β-Ni(OH)_2_ on the MWNTs was *~*2 nm. In addition, independent β-Ni(OH)_2_ films were observed between the MWNTs (Figs 2h and S1b). The PyPBI layer on the MWNTs was also dominant for the formation of the fine nanostructure of MWNT-PyPBI-Ni(OH)_2_. Without using PyPBI, a very limited surface area of the bare MWNTs was covered by the β-Ni(OH)_2_ particles after the same synthetic procedure (Figure S4).

The synthesized catalysts were further characterized by X-ray diffraction analysis (XRD), Raman spectroscopy, and X-ray photoelectron spectroscopy (XPS). In all XRD patterns (Fig. 3a), the peak that appeared at 26.1° is related to the (002) faces of the MWNTs^29^. Besides, the phases of the spinel oxides are observed for the MWNT-PyPBI-Ni_*x*_Co_3−*x*_O_4_ and MWNT-PyPBI-Co_3_O_4_, while β-Ni(OH)_2_ is detected for the MWNT-PyPBI-Ni(OH)_2_, which well agrees with the TEM results shown in Fig. 2^30^. The crystallinity of the MWNTs in the catalysts were assessed by the *I*_*G*_/*I*_*D*_ values in the Raman spectra, in which *I*_*G*_ and *I*_*D*_ refer to the intensities of the sp^2^ and sp^3^ vibrations, respectively. Higher *I*_*G*_/*I*_*D*_ values represent a better crystallinity of the graphitic carbon structure. Figure 3b shows that the *I*_*G*_/*I*_*D*_ values of the solvothermally-treated products are even slightly higher than that of the as-prepared MWNT-PyPBI, demonstrating that the MWNTs were not damaged. Instead, the graphitic crystallinity was improved during the solvothermal treatment^31^,^32^. As shown in Fig. 3c, XPS peaks appeared at 400.3 eV and 398.7 eV, which are derived from the pyrrolic N and pyridinic N of PyPBI, respectively. Such peaks appeared for all three samples, indicating that PyPBI remained in unchanged structure even after the solvothermal treatment^25^. Finally, the expected metallic elements were detected in the XPS spectra of all the samples (see Fig. 3c). For MWNT-PyPBI-Ni_*x*_Co_3−*x*_O_4_, the peaks of Co 2p were mainly related to Co^3+ ^33, and the Ni 2p peaks were attributed to Ni^2+^ combined with O^34^. For the MWNT-PyPBI-Ni(OH)_2_, the Ni 2p peaks shifted to a higher band energy compared to those of the MWNT-PyPBI-Ni_*x*_Co_3−*x*_O_4_ and can be attributed to Ni(OH)_2_^34^. For MWNT-PyPBI-Co_3_O_4_, the coexistence of Co^2+^ and Co^3+^ was recognized^35^. The above results suggested that we have succeeded in coating the PyPBI-wrapped MWNTs with uniform Ni/Co spinel oxide nanoparticles to provide the MWNT-PyPBI-Ni_*x*_Co_3−*x*_O_4_. To the best of our knowledge, such a fine composite nanostructure with ~5nm-size of the Ni/Co spinel oxide on the undamaged high crystalline MWNTs has not yet been reported.

Based on the obtained results, a mechanism for the formation of the nanostructures of the three samples is suggested as follows. PyPBI homogeneously wraps the MWNTs via strong π-π interactions^22^. As a result, the PyPBI layer on the nanotubes provides well-dispersed binding sites, which coordinate with the transition metal cations (Co^2+^ and Ni^2+^) during the syntheses^22^. Subsequently, transition metal oxides or hydroxide are *in-situ* synthesized by the solvothermal treatment. For the MWNT-PyPBI-Co_3_O_4_, Co_3_O_4_ nanoparticles tend to aggregate due to the fast growth of the crystal nuclei^28^, resulting in the formation of large secondary particles on the MWNTs. On the other hand, nanosized β-Ni(OH)_2_ particles are well coated on the MWNT-PyPBI and no agglomeration occurs. Such agglomeration-free structure of β-Ni(OH)_2_ nanosheets resulted from solvothermal synthesis has also been reported by other group^36^. Obviously, the presence of Ni is responsible for the immobilization of the Ni/Co spinel oxide nanoparticles on the PyPBI layer during the solvothermal treatment, resulting in the homogeneous distribution of uniform Ni/Co spinel oxide nanoparticles on the MWNT-PyPBI. However, further investigation on the formation mechanism of these Ni/Co spinel oxide nanoparticles during solvothermal synthesis is needed to clarify the detailed mechanism.

The electrocatalytic activities of the catalysts for the OER and ORR were measured by linear sweep voltammetry (LSV) using a rotating ring-disk electrode in an O_2_-saturated 1 M KOH solution. The IR-compensated results are shown in Fig. 4 and the uncompensated results are shown in Figure S4. Since the ohmic resistances of the electrodes determined by electrochemical impedance spectroscopy are as small as 0.8~1.2 Ω cm^2^ (see Figure S5), the ORR profiles with and without IR compensation are almost the same while the OER results are different due to the large current density. For OER (see Fig. 4a), the potential at 10 mA cm^−2^ and Tafel slope of MWNT-PyPBI-Ni_*x*_Co_3−*x*_O_4_ were respectively *ca*. 1.54 V and 42 mV dec^−1^, comparable to the performance of IrO_2_/C (1.53 V and 56 mV dec^−1^) and the state-of-art OER catalysts in the literature (see Supplementary, Table S2)^37^,^38^,^39^,^40^,^41^,^42^. An oxidation current peak at *ca*. 1.305 V vs. RHE was observed for the MWNT-PyPBI-Ni_*x*_Co_3−*x*_O_4_, which can be ascribed to the Ni^3+^/Ni^2+^ redox couple of NiCo_2_O_4_^35^. We consider that the pristine MWNTs provide efficient electron conducting paths for the Ni_*x*_Co_3−*x*_O_*y*_ nanoparticles, which promotes the electron transfer process and thus improve the OER activity of the MWNT-PyPBI-Ni_*x*_Co_3−*x*_O_4_. This mechanism also worked for the MWNT-PyPBI-Ni(OH)_2_; namely, the potential at 10 mA cm^−2^ of the catalyst was 1.56 V and the Tafel slope was as low as 68 mV dec^−1^. Such good OER properties are unusual because β-Ni(OH)_2_ has not yet been reported as a good OER catalyst in the literature; for example, Gao *et al*. reported an OER Tafel slope of 246 mV dec^−1^ for β-Ni(OH)_2_ nanoparticle^43^. The poor electrical conductivity of β-Ni(OH)_2_ is one of the key limiting factors for their poor electrocatalytic activity^44^,^45^. For the MWNT-PyPBI-Ni(OH)_2_, however, the β-Ni(OH)_2_ layer on the MWNTs was extremely thin (Fig. 2g) and the pristine MWNTs possesses high electrical conductivity, which would be responsible for the improved OER activity of the MWNT-PyPBI-Ni(OH)_2_. On the other hand, the OER current density of the MWNT-PyPBI-Co_3_O_4_ was much poorer than that of the MWNT-PyPBI-Ni_*x*_Co_3−*x*_O_4_, which should be due to the low electrical conductivity and agglomeration of Co_3_O_4_ nanoparticles on the MWNTs.

The ORR performance of the catalysts is shown in Fig. 4c–e. The potentials at 0.05 mA cm^−2^ in the ORR LSV profiles are recognized as the onset potentials. The MWNT-PyPBI-Ni_*x*_Co_3−*x*_O_4_ has a similar onset potential (*E*_*onset,ORR*_: 0.918 V vs. RHE), but more positive half-wave potential (*E*_*1/2*_: 0.811 V vs. RHE) and smaller Tafel slope (55 mV dec^−1^) compared to the MWNT-PyPBI-Co_3_O_4_ (*E*_*onset,ORR*_: 0.912 V vs. RHE; *E*_*1/2*_: 0.775 V vs. RHE; Tafel slope: 73 mV dec^−1^). The improved electrical conductivity and nanostructure due to the incorporation of Ni into Co_3_O_4_ and the hybridizing with pristine MWNTs should be responsible for the enhanced ORR performance^12^. It is worth noting that the ORR performance of MWNT-PyPBI-Ni_*x*_Co_3−*x*_O_4_ is close to commercial Pt/C (*E*_*onset,ORR*_: 0.951 V vs. RHE; *E*_*1/2*_: 0.857 V vs. RHE; Tafel slope: 57 mV dec^−1^) and superior to the state-of-art cobalt-based ORR catalysts reported in the literature (Table S3)^13^,^14^,^41^,^46^,^47^. It is considered that the pristine MWNTs form an efficient electron transport network and the well-constructed nanostructure of Ni_*x*_Co_3−*x*_O_4_ on the MWNT-PyPBI provides a large surface area, leading to the enhanced electrocatalytic performance. Moreover, Fig. 4e shows that the Koutecky-Levich plots of MWNT-PyPBI-Ni_*x*_Co_3−*x*_O_4_, MWNT-PyPBI-Co_3_O_4_, and Pt/C based on their LSV curves measured at different rotation rates exhibit very close slopes (see Figure S6), demonstrating that the 4-electron reaction process dominates the ORR on MWNT-PyPBI-Ni_*x*_Co_3−*x*_O_4_ and MWNT-PyPBI-Co_3_O_4_^48^.

The durability of the MWNT-PyPBI-Ni_*x*_Co_3−*x*_O_4_ and precious metal catalysts (Pt/C and IrO_2_/C) was investigated by chronopotentiometry tests. For OER and ORR, the catalysts were respectively held under constant potentials of 1.555 V and 0.805 V vs. RHE and the current density was recorded as a function of time (Figure S7). For the convenience of comparison, the relative current of the catalysts was plotted against time in Fig. 5. For OER, Fig. 5a shows that throughout the chronopotentiometry test, no decrease in relative current was observed for MWNT-PyPBI-Ni_*x*_Co_3−*x*_O_4_ while that for IrO_2_/C decreased to 33%. On the other hand, MWNT-PyPBI-Ni_*x*_Co_3−*x*_O_4_ exhibited better durability for ORR than Pt/C. Figure 5b shows that 79% of current was remained after the chronopotentiometry test. These results have clearly demonstrated that the MWNT-PyPBI-Ni_*x*_Co_3−*x*_O_4_ is very durable and an efficient catalyst as a bifunctional catalyst.

In summary, we have succeeded in the preparation of a bifunctional (ORR and OER) catalyst by decorating pristine MWNTs with uniform nanoparticles of a Ni_*x*_Co_3−*x*_O_4_ spinel oxide with the assistance of PyPBI. Based on the characterization results, a possible mechanism for the formation of the composite nanostructure of the MWNTs, PyPBI, and Ni_*x*_Co_3−*x*_O_4_ nanoparticles was proposed. Based on such a fine nanostructure, the MWNT-PyPBI-Ni_*x*_Co_3−*x*_O_4_ exhibited excellent activities and durability towards both OER and ORR. Finally, we like to emphasize that this facile strategy for preparing the hybrid catalyst of Ni_*x*_Co_3−*x*_O_4_ and pristine MWNTs provides an easy scalability and can be applicable to other transition metal oxides, for example, peroviskites with high OER activity but low electrical conductivity^49^. Such studies are now in progress in our laboratory.

## Methods

### Materials

MWNTs (*ϕ* ~ 20 nm) were kindly provided by Nikkiso Co., Ltd. PyPBI was synthesized according to a previous report^26^. Nickel acetylacetonate, cobalt acetylacetonate, and a 5 wt% Nafion solution in a mixture of lower aliphatic alcohols and water were purchased from Sigma-Aldrich Co. LLC. Carbon black (XC-72R) was purchased from the Fuel Cell Store. IrCl_3_·*x*H_2_O was purchased from Tokyo Chemical Industry Co., Ltd. N,N’-dimethylacetamide (DMAc) was purchased from Kishida Chemical Co., Ltd. Ethanol, ammonia solution, 2-Propanol, and ethylene glycol (EG) were purchased from Wako Pure Chemical Industries, Ltd. Pt/C commercial powder (Pt 37 wt%) was purchased from Tanaka Kikinzoku Kogyo Co., Ltd. All the chemicals were used as received.

### Preparation of catalysts

The MWNTs (15 mg) were mixed with PyPBI (5 mg) in DMAc (20 mL) and sonicated for 6 h to form PyPBI-wrapped MWNTs (MWNT-PyPBI). Nickel acetylacetonate (8.8 mg), cobalt acetylacetonate (17.5 mg), and MWNT-PyPBI (10 mg) were then sonicated in ethanol (30 mL), water (1.9 mL), and ammonia water (0.6 mL) for 15 min. The obtained suspension was then refluxed at 80 °C for 20 h, and subsequently transferred to a Teflon autoclave (60 mL) for a solvothermal treatment at 150 °C for 3 h. The obtained composite catalyst was denoted MWNT-PyPBI-Ni_*x*_Co_3−*x*_O_4_. For comparison, nickel acetylacetonate (22.6 mg) or cobalt acetylacetonate (26.3 mg) was separately used following the same preparation procedure, and the products were denoted as MWNT-PyPBI-Ni(OH)_2_ and MWNT-PyPBI-Co_3_O_4_, respectively. In order to clarify the role of PyPBI, bare MWNTs were also used as the support for the Ni/Co oxide and Ni(OH)_2_. Ir/carbon black (Ir/C) was prepared by refluxing IrCl_3_·*x*H_2_O (7.9 mg) and carbon black (10 mg) in 60% ethylene glycol aqueous solution (20 mL) at 160 °C for 6 h under an N_2_ atmosphere. The formation of Ir nanoparticles on carbon black was confirmed by X-ray diffraction analysis (see Figure S8) and the Ir content in Ir/C was estimated to be 15.4 wt% by thermogravimetric analysis.

### Characterization

The microstructures of the catalysts were observed using an SU9000 (Hitachi High-Technologies) operated at 30 kV and a JEM-ARM200CF (JEOL) operated at 120 kV. The amount of metallic species in the catalysts were estimated by thermogravimetric analysis (TG, EXSTAR TG/DTA6300, SEIKO Instruments, Inc). The crystal structure was characterized by X-ray diffraction analysis (XRD, SmartLab, Rigaku Corp.). The electron conditions were determined by X-ray photoelectron spectroscopy (XPS, AXIS-ULTRA, Shimazu Corp.). The crystallinity of the MWNTs was evaluated by Raman spectroscopy (533 nm, Raman-touch, Nanophoton Corp.).

### Electrochemical evaluation

The electrocatalytic activities for the OER and ORR were evaluated in a three-electrode system. 0.82 mg sample of each catalyst (MWNT-PyPBI-Ni_*x*_Co_3−*x*_O_4_, MWNT-PyPBI-Co_3_O_4_, and MWNT-PyPBI-Ni(OH)_2_) was dispersed in a mixed solution of 2-propanol (336 μL), H_2_O (84 μL), and Nafion solution (6 μL) to form a catalyst ink. A 20 μl aliquot of the ink was casted on the glassy carbon electrode of a rotating ring-disk electrode (RRDE, *ϕ* 4 mm, BAS Inc.) and air-dried, resulting in a catalyst loading of 0.3 mg cm^−2^. For comparison, a Pt/C electrode with a Pt loading of 14.2 μg_Pt_ cm^−2^ and an Ir/carbon black electrode with a Ir loading of 10.2 μg_Ir_ cm^−2^ were prepared in the same way. Prior to the OER evaluation, the Ir/carbon black electrode was electrochemically oxidized to IrO_2_/carbon black (IrO_2_/C) electrode by cyclically sweep the potential between 1.1 V and 1.5 V vs. RHE^50^. A 1 M KOH solution, Ag/AgCl in saturated KCl solution, and Pt wire were used as the electrolyte, reference electrode, and counter electrode, respectively. The potential vs. Ag/AgCl was converted to the reversible hydrogen electrode (RHE) scale by:


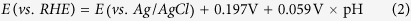


The linear sweep voltammetry, cyclic voltammetry, and impedance spectroscopy measurements were conducted by a potentiostat (ALS 760D, ALS Co., Ltd.) at room temperature. The data of the electrochemical tests were IR-compensated. Chronopotentiometry tests were conducted to investigate the durability of the electrocatalsyts for ORR and OER. For OER, MWNT-PyPBI-Ni_*x*_Co_3−*x*_O_4_ and Pt/C were held at a constant potential of 1.54 V vs. RHE and the current density was recorded against time. For OER, MWNT-PyPBI-Ni_*x*_Co_3−*x*_O_4_ was compared with IrO_2_/C and the constant potential was fixed to be 0.805 V vs. RHE. All the results of the electrochemical tests have been proved to be reproducible.

## Additional Information

**How to cite this article**: Yang, J. *et al*. Decorating unoxidized-carbon nanotubes with homogeneous Ni-Co spinel nanocrystals show superior performance for oxygen evolution/reduction reactions. *Sci. Rep.* **7**, 45384; doi: 10.1038/srep45384 (2017).

**Publisher's note:** Springer Nature remains neutral with regard to jurisdictional claims in published maps and institutional affiliations.

## Supplementary Material

Supplementary Information

## Figures and Tables

**Figure 1:**
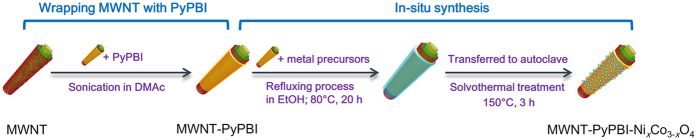
Preparation procedure of MWNT-PyPBI-Ni_*x*_Co_3−*x*_O_4_. MWNTs were wrapped with PyPBI, and then Ni_*x*_Co_3−*x*_O_4_ was *in-situ* synthesized by a facile solvothermal treatment.

**Figure 2:**
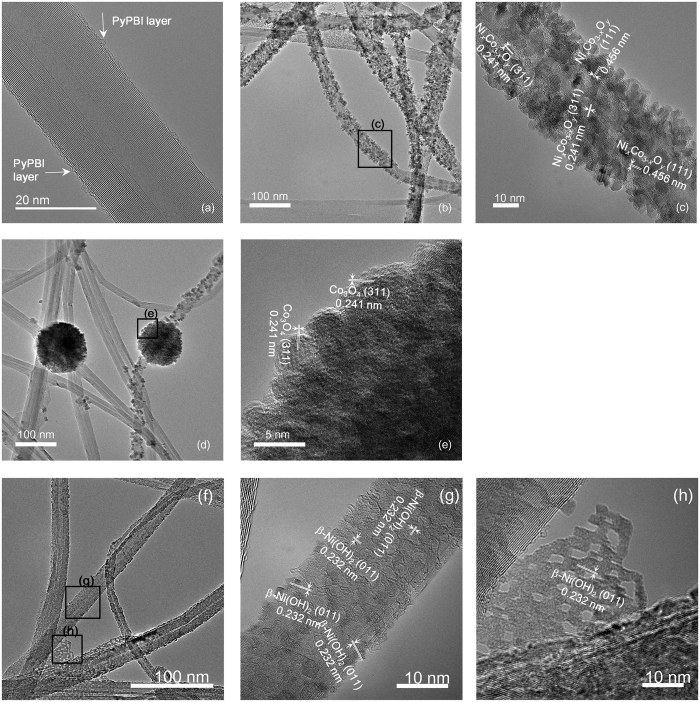
Morphologies of metal oxides deposited on MWNT-PyPBI. TEM images of (**a**) MWNT-PyPBI, (**b**,**c**) MWNT-PyPBI-Ni_*x*_Co_3−*x*_O_4_, (**d**,**e**) MWNT-PyPBI-Co_3_O_4_, and (**f**,**g**,**h**) MWNT-PyPBI-Ni(OH)_2_; the TEM images of the black rectangle-selected area in (**b**,**d**,**f**) obtained in larger magnification are also shown in (**c**,**e**,**g**,**h**).

**Figure 3:**
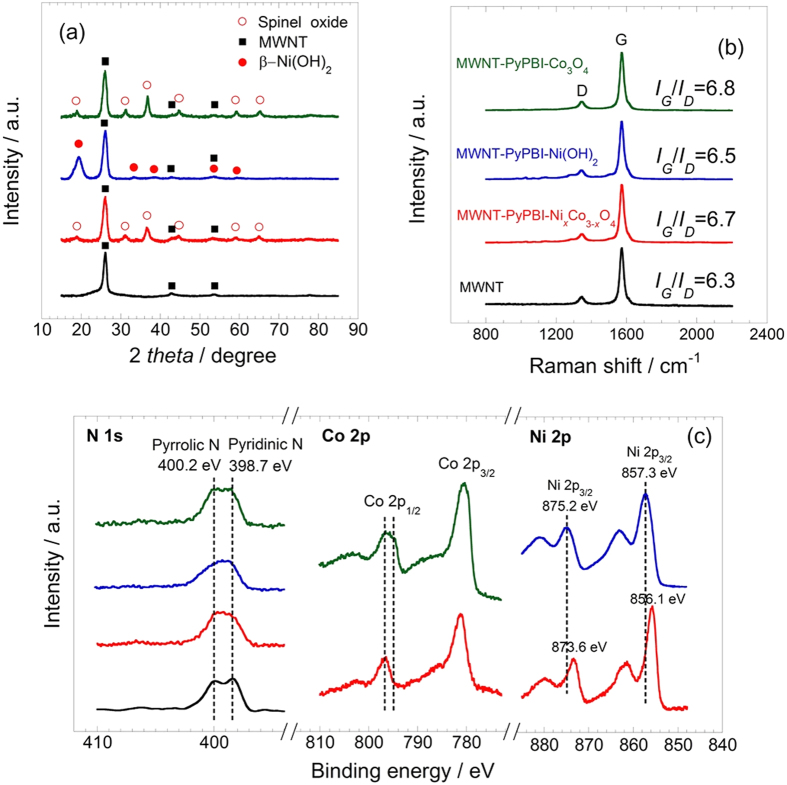
Structure identification of the three products. (**a**) XRD patterns, (**b**) Raman spectra, and (**c**) XPS profiles of MWNT-PyPBI-Ni_*x*_Co_3−*x*_O_4_ (red), MWNT-PyPBI-Co_3_O_4_ (green), MWNT-PyPBI-Ni(OH)_2_ (blue), and MWNT (black).

**Figure 4:**
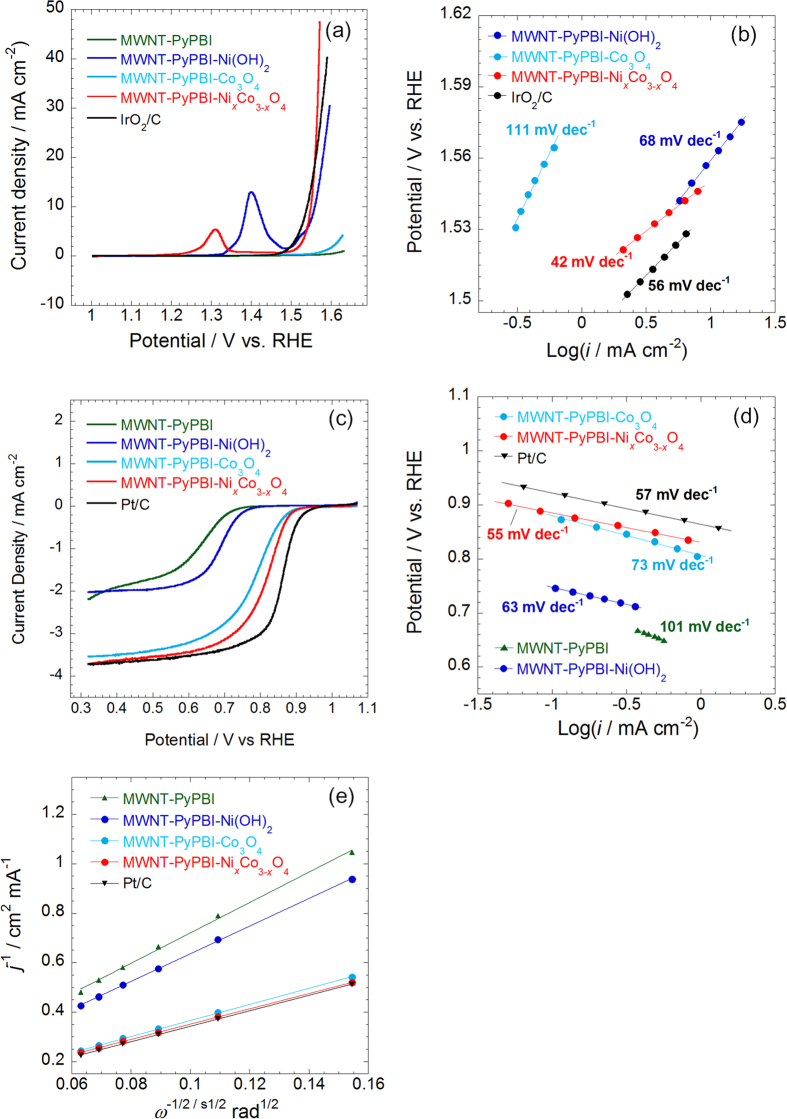
Evaluation of the bifunctional catalysts. (**a**) OER polarization curves, (**c**) ORR polarization curves, and (**e**) K-L plots of MWNT-PyPBI, MWNT-PyPBI-Ni(OH)_2_, MWNT-PyPBI-Co_3_O_4_, and MWNT-PyPBI-Ni_*x*_Co_3−*x*_O_4_; the ORR and K-L curves of Pt/C and the OER curve of IrO_2_/carbon black are also presented for comparison; the corresponding Tafel plots are shown in (**b**,**d**); electrolyte: O_2_-saturated 1 M KOH solution; scan rate: 10 mV s^−1^; rotating rate: 1600 rpm; catalyst loading for non-platinum catalysts: 0.3 mg cm^−2^; catalyst loading for Pt/C: 14.2 mg_Pt_ cm^−2^; catalyst loading for IrO_2_/C: 10.2 mg_Pt_ cm^−2^; temperature: 25 °C.

**Figure 5:**
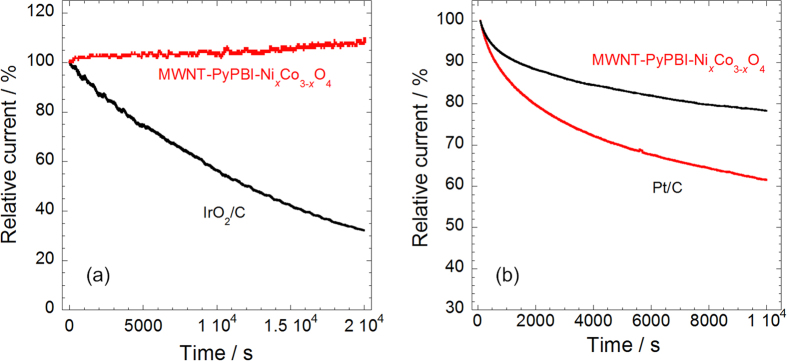
Long-term stability of MWNT-PyPBI-Ni_*x*_Co_3−*x*_O_4_ as a bifunctional catalyst. (**a**) Relative current of MWNT-PyPBI-Ni_*x*_Co_3−*x*_O_4_ and Pt/C held at a constant potential of 1.54 V vs. RHE; (**b**) the current densities of MWNT-PyPBI-Ni_*x*_Co_3−*x*_O_4_ and IrO_2_/C held at a constant potential of 0.805 V vs. RHE; electrolyte: O_2_-saturated 1 M KOH; temperature: 25 °C.
