# Supplementary material for: Decorating unoxidized-carbon nanotubes with homogeneous Ni-Co spinel nanocrystals show superior performance for oxygen evolution/reduction reactions
Source: Sci Rep. 2017 Mar 30;7:45384. doi: 10.1038/srep45384 (PMC5371823; doi:10.1038/srep45384)
Supplement: Supplementary Information [file srep45384-s1.pdf]

Supplementary Information for

Decorating unoxidized-carbon nanotubes with homogeneous Ni-Co spinel nanocrystals show superior performance for oxygen evolution/reduction reactions

Jun Yang,[1,2] Tsuyohiko Fujigaya,[1,2,3] and Naotoshi Nakashima[1,2,]*

[1] International Institute for Carbon-Neutral Energy Research (WPI-I^{2}CNER), Kyushu University, 744 Motooka, Nishi-ku, Fukuoka 819-0395, Japan

[2] Department of Applied Chemistry, Graduate School of Engineering, Kyushu University, 744 Motooka, Nishi-ku, Fukuoka 819-0395, Japan

[3] PRESTO, JST, 4-1-8 Honcho, Kawaguchi, Saitama, 332-0012, Japan

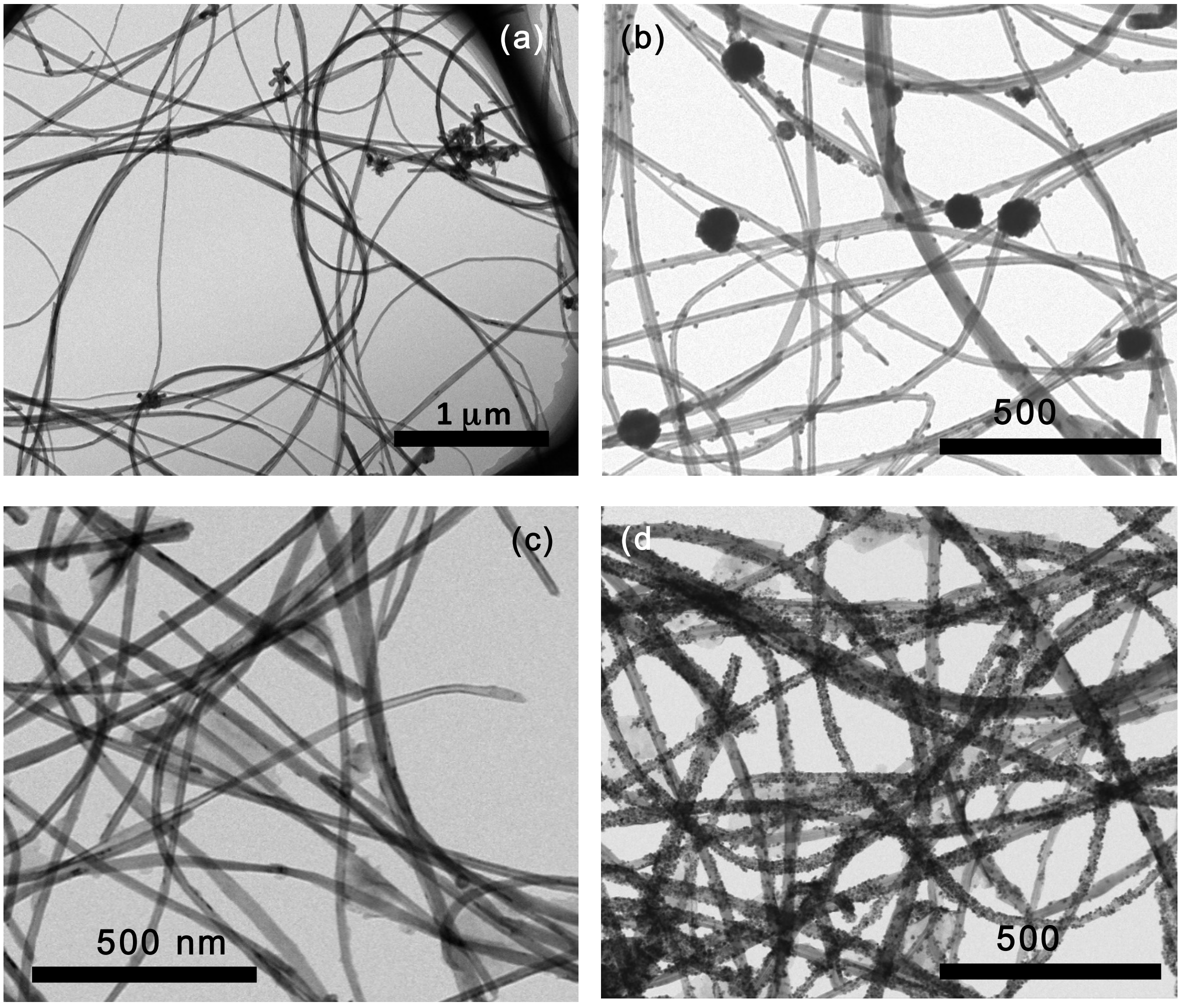

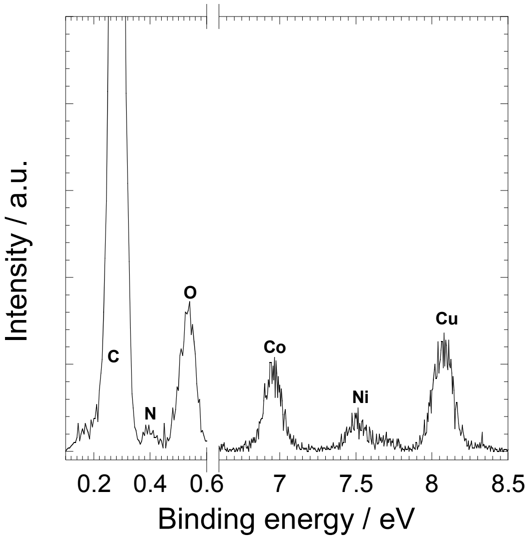

Figure S1. STEM images of (a) MWNT-PyPBI, (b) MWNT-PyPBI-Co_3O_4, (c) MWNT-PyPBI-$Ni(OH)_2$, and (d) MWNT-PyPBI-$Ni_xCo_{3-x}O_4$ at lower magnification than Figure 2; (e) EDS analysis result for the region of (d).

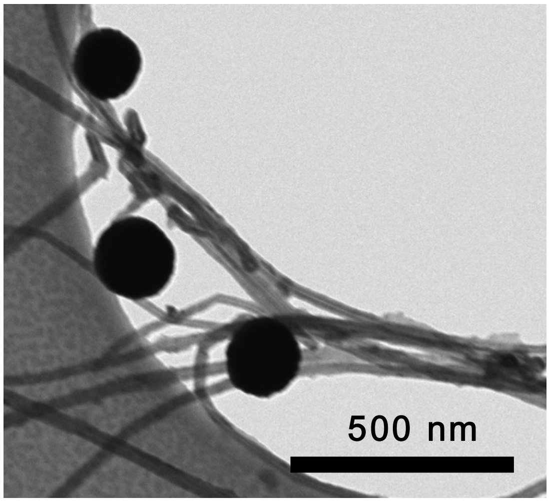

Figure S2. STEM images of MWNT-PyPBI-$Ni_xCo_{3-x}O_4$ prepared without PyPBI.

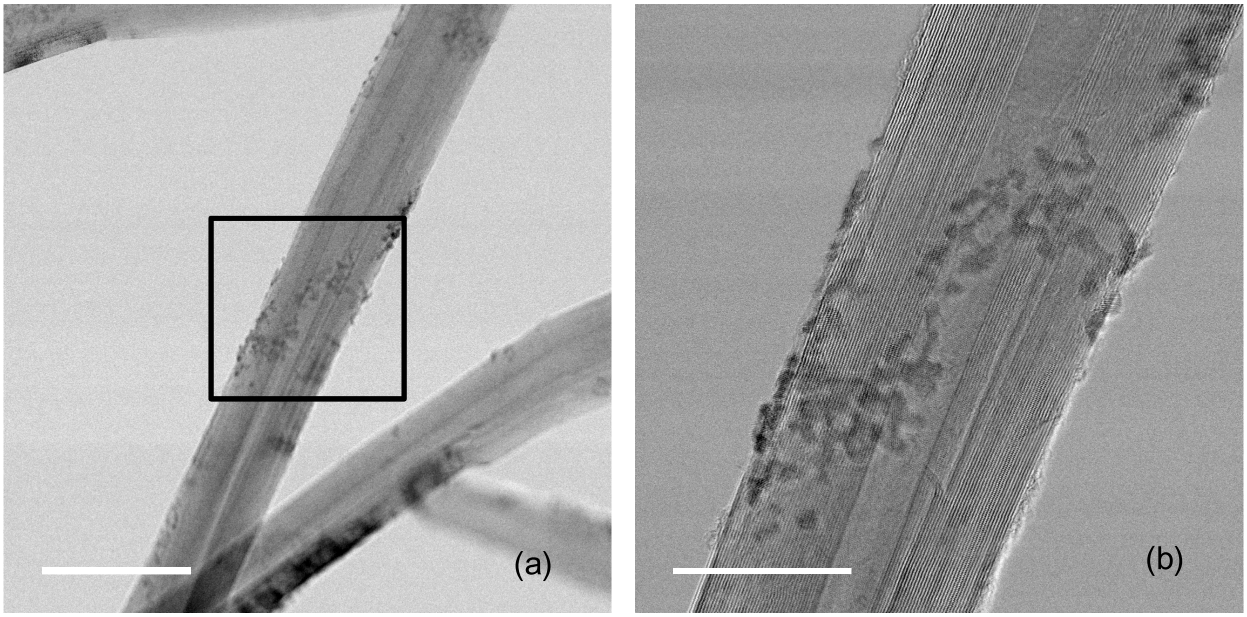

Figure S3. (a) TEM images of MWNT-PyPBI-Ni$(OH)_2$ prepared without PyPBI; (b) enlarged image of the selected area in (a).

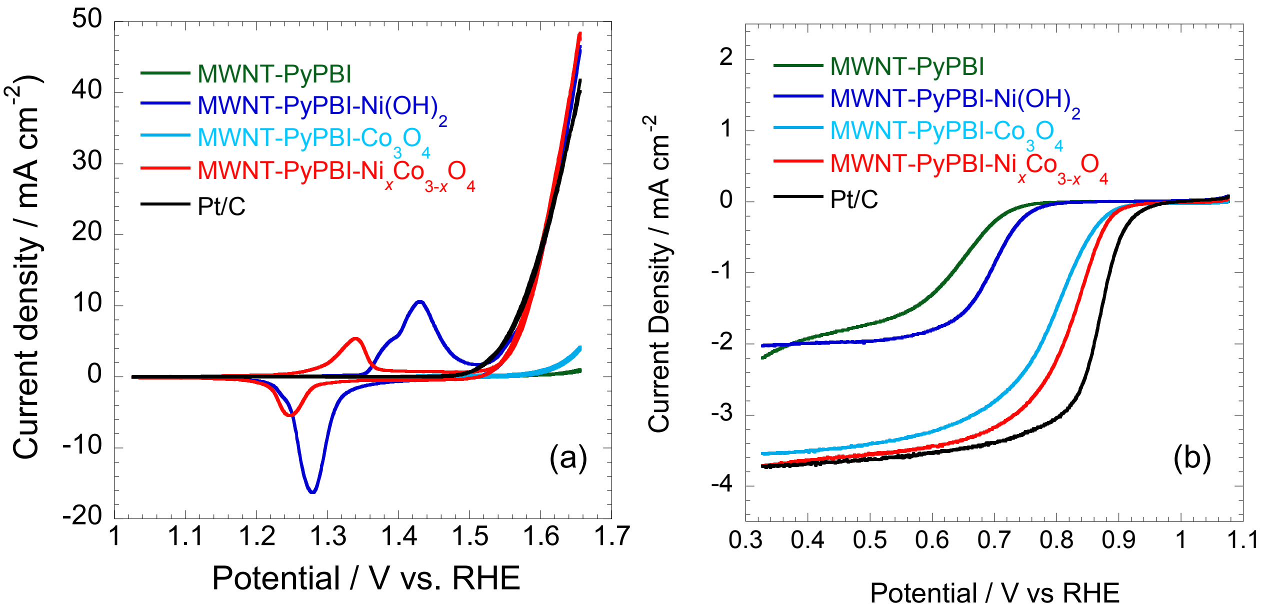

Figure S4. (a) OER polarization curves and (b) ORR polarization curves MWNT-PyPBI, MWNT-PyPBI-$Ni(OH)_2$, MWNT-PyPBI-Co_3O_4, and MWNT-PyPBI-$Ni_xCo_{3-x}O_4$ without IR compensation; the ORR and curve of Pt/C and the OER curve of IrO_2/carbon black are also presented for comparison; electrolyte: O_2-saturated 1 M KOH solution; scan rate: 10 mV s^{-1}; rotating rate: 1600 rpm; catalyst loading for non-platinum catalysts: 0.3 mg cm^{-2}; catalyst loading for Pt/C: 14.2 mg_{Pt} cm^{-2}; catalyst loading for IrO_2/C: 10.2 mg_{Pt} cm^{-2}; temperature: 25 °C.

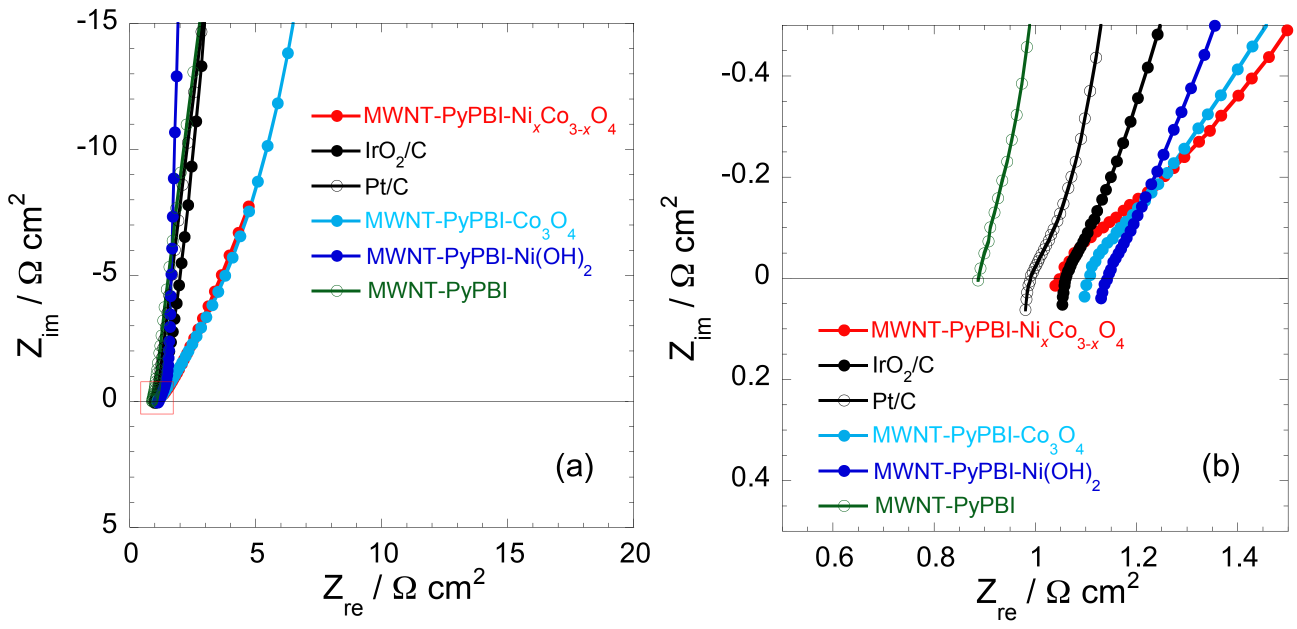

Figure S5. (a) Impedance spectra of MWNT-PyPBI, MWNT-PyPBI-Ni(OH)$_2$, MWNT-PyPBI-Co$_3$O$_4$, MWNT-PyPBI-Ni$_x$Co$_{3-x}$O$_4$, Pt/C, and IrO$_2$/carbon black; (b) enlarged image of the region in (a); electrolyte: O$_2$-saturated 1 M KOH solution; scan rate: 10 mV s^{-1}; catalyst loading for non-platinum catalysts: 0.3 mg cm^{-2}; catalyst loading for Pt/C: 14.2 mg$_{Pt}$ cm^{-2}; catalyst loading for IrO$_2$/C: 10.2 mg$_{Ir}$ cm^{-2}; temperature: 25 °C.

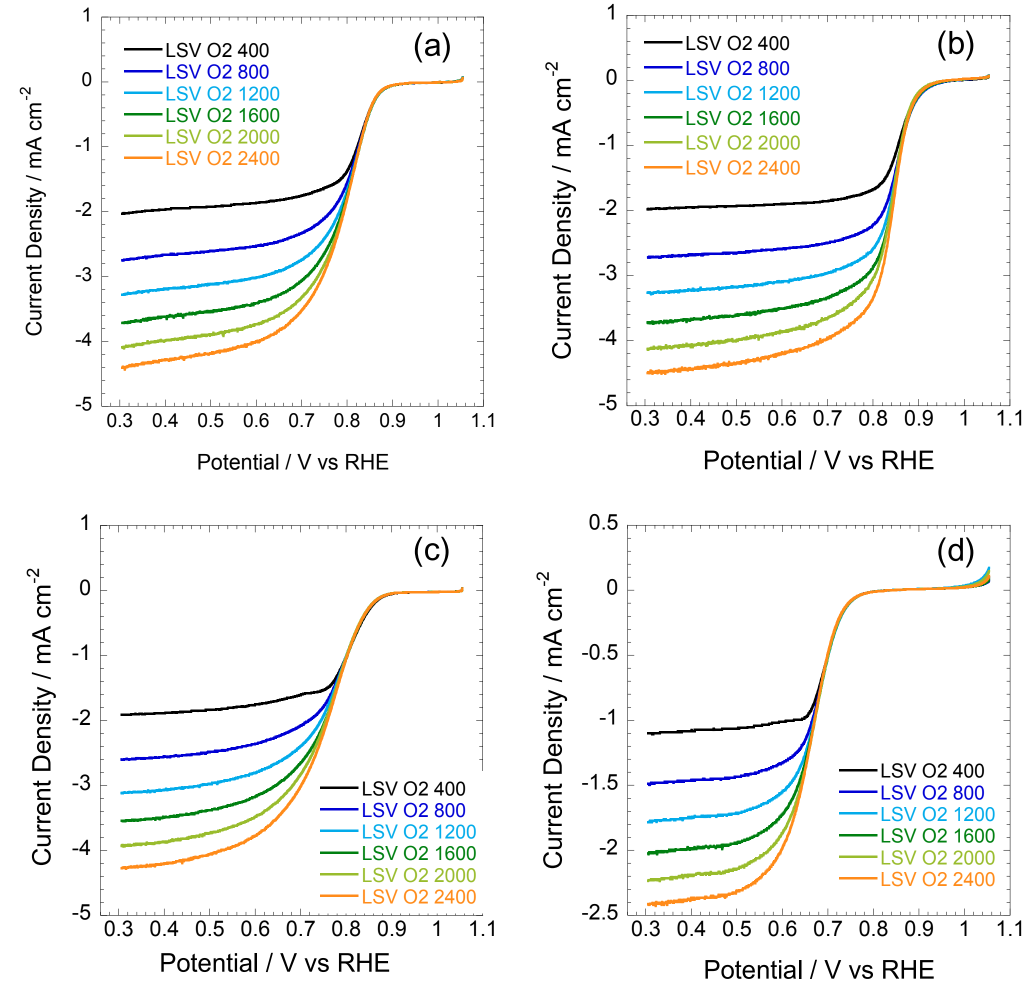

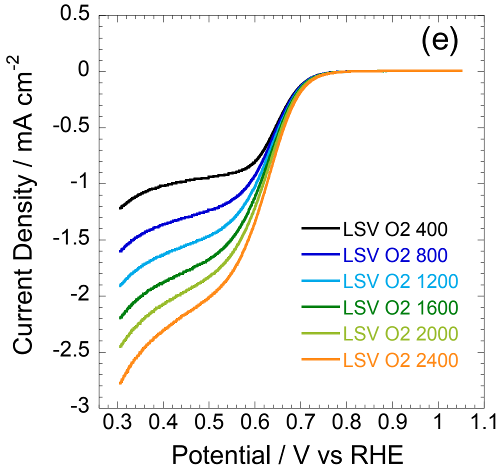

Figure S6. LSV profiles of (a) MWNT-PyPBI-$Ni_xCo_{3-x}O_4$, (b) Pt/C, (c) MWNT-PyPBI-Co_3O_4, (d) MWNT-PyPBI-$Ni(OH)_2$, and (e) MWNT-PyPBI measured at 10 mV s^{-1} in O_2-saturated 1 M KOH with different rotation rates indicated.

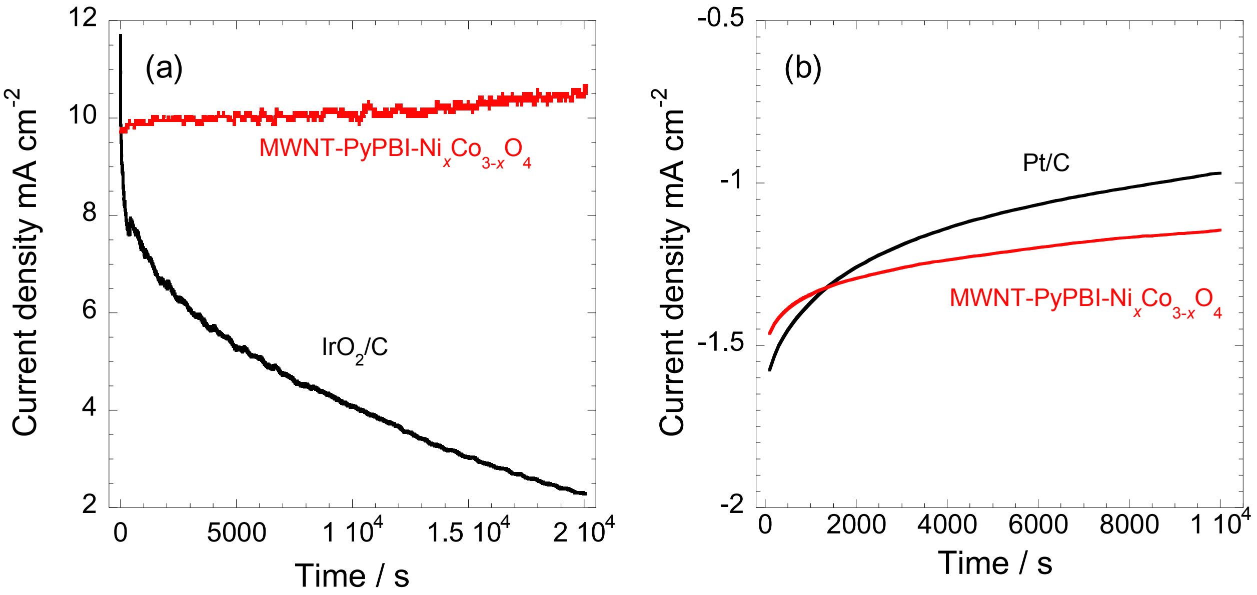

Figure S7. (a) The current densities of MWNT-PyPBI-$Ni_xCo_{3-x}O_4$ and Pt/C held at a constant potential of 1.54 V vs. RHE; (b) the current densities of MWNT-PyPBI-$Ni_xCo_{3-x}O_4$ and IrO_2/C held at a constant potential of 0.805 V vs. RHE; electrolyte: O_2-saturated 1 M KOH; temperature: 25 ^{o}C.

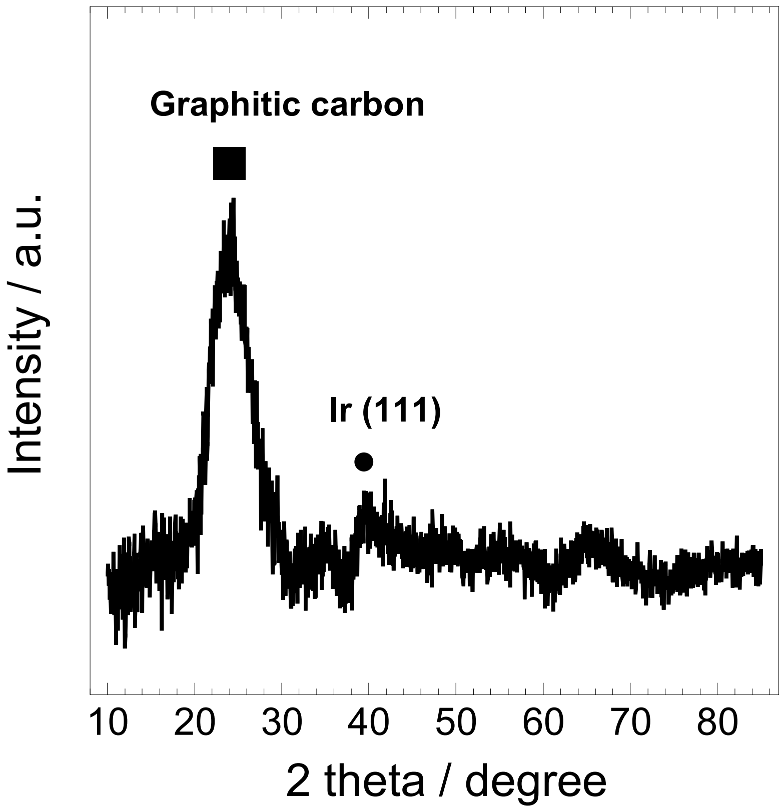

Figure S8. X-ray diffraction pattern of Ir/C.

Table S1. Atomic ratio of elements of MWNT-PyPBI-$Ni_xCo_{3-x}O_4$ estimated from the EDS result in Figure S1e.

Atomic ratio of elements / %				
C	N	O	Co	Ni
84.4	1.9	11.4	1.6	0.7

Table S2. Comparison of the OER activities of non-precious metal-based catalysts in 1 M or 0.1 M KOH solution.

Samples	E_{OER} @ 10mA cm^{-2} (V vs. RHE)	Tafel slope (mV dec^{-1})	Reference
MWNT-PyPBI-$Ni_xCo_{3-x}O_4$	1.59	37	This work
Ni/NiO/$NiCo_2O_4$/N-CNT-As	1.60	77	15
$NiCo_2O_4$ hollow microcuboids	1.52	53	34
$NiCo_2O_4$ nanowire array	1.77	90	35
mesoporous $NiCo_2O_4$ nanocage	1.54	75	36
Co_3O_4/$NiCo_2O_4$ Double-Shelled Nanocage	1.57	88	37
NCNT/CoO-NiO-NiCo alloy	1.52	40	38
Nickel–Cobalt Binary Oxide Nanoporous Layers	1.56	39	39

Table S3. Comparison of the ORR activities of non-precious metal-based catalysts in 1 M or 0.1 M KOH solution.

Samples	Electrolyte	$E_{onset,ORR}$ (V vs. RHE)	$E_{1/2}$ (V vs. RHE)	Tafel slope (mV dec^{-1})	Reference
MWNT-PyPBI-$Ni_xCo_{3-x}O_4$	1 M KOH	0.918	0.811	55	This work
Pt/C	1 M KOH	0.951	0.857	57	This work
$NiCo_2O_4$/PVP/graphene	0.1 M KOH	0.912	0.777	-	13
Co-CoO/N-rGO	0.1 M KOH	0.880	0.780	40	14
NCNT/CoO-NiO-NiCo alloy	1 M KOH	1.000	0.830	63	38
N-CG-CoO	1 M KOH	0.90	0.810	48	43
(Ni,Co)/CNT	0.1 M KOH	0.901	0.751	-	44
